# Determinants of Processing Speed Trajectories among Middle Aged or Older Adults, and Their Association with Chronic Illnesses: The English Longitudinal Study of Aging

**DOI:** 10.3390/life11040357

**Published:** 2021-04-18

**Authors:** Viktor Gkotzamanis, Giorgos Koliopanos, Albert Sanchez-Niubo, Beatriz Olaya, Francisco Félix Caballero, José Luis Ayuso-Mateos, Somnath Chatterji, Josep Maria Haro, Demosthenes Panagiotakos

**Affiliations:** 1 School of Health Science and Education, Harokopio University, 17671 Athens, Greece; vik.gkot@hua.gr (V.G.); koliopanos92@gmail.com (G.K.); 2 Parc Sanitari Sant Joan de Déu, Universitat de Barcelona, 08830 Sant Boi de Llobregat, Spain; albert.sanchez@pssjd.org (A.S.-N.); beatriz.olaya@pssjd.org (B.O.); jmharo@pssjd.org (J.M.H.); 3 Department of Preventive Medicine and Public Health, School of Medicine, Universidad Autónoma de Madrid, 28029 Madrid, Spain; felix.caballero@uam.es; 4 CIBER of Mental Health, 28007 Madrid, Spain; 5 CIBER of Epidemiology and Public Health, 28029 Madrid, Spain; 6 Department of Psychiatry, Universidad Autónoma de Madrid, 28029 Madrid, Spain; joseluis.ayuso@uam.es; 7 Hospital Universitario de La Princesa, Instituto de Investigación Sanitaria Princesa (IP), 28006 Madrid, Spain; 8 Instituto de Salud Carlos III, Centro de Investigación Biomédica en Red de Salud Mental, CIBERSAM, 28029 Madrid, Spain; 9 Information, Evidence and Research, World Health Organization, 1202 Geneva, Switzerland; chatterjis@who.int

**Keywords:** aging, processing speed, ELSA, longitudinal analysis

## Abstract

The aim of this study was to identify latent groups of similar trajectories in processing speed through aging, as well as factors that are associated with these trajectories. In the context of the Ageing Trajectories of Health: Longitudinal Opportunities and Synergies (ATHLOS) project, data from the English Longitudinal Study of Aging (ELSA) (n = 12099) were analyzed. Latent groups of similar trajectories in the processing scores as well as their predictors and covariates were investigated, using group-based trajectory models (GBTM). The coefficient estimates for potential group predictors correspond to parameters of multinomial logit functions that are integrated in the model. Potential predictors included sex, level of education, marital status, level of household wealth, level of physical activity, and history of smoking, while time-varying covariates included incidence of cardiovascular disease (CVD), diabetes mellitus, depressive symptoms, and sleep disturbances. Four trajectories were identified and named after their baseline scores and shapes: High (4.4%), Middle/Stable (31.5%), Low/Stable (44.5%), and Low Decline (19.6%). Female sex, higher levels of education, mild level of physical activity, having been married, and higher level of wealth were associated with a higher probability of belonging to any of the higher groups compared to the Low/Decline that was set as reference, while presence of CVD, diabetes mellitus, and depressive symptoms were associated with lower processing speed scores within most trajectories. All the aforementioned factors might be valid targets for interventions to reduce the burden of age-related cognitive impairment.

## 1. Introduction

Ageing is associated with deterioration in cognitive function. This decline is in turn associated with difficulties in performing tasks of everyday living, leading ultimately to disability and dependence. Perseverance of cognitive abilities, through advancing age, is of great importance for individuals to sustain a good quality of life, as well as for societies and public health systems, especially in a setting of population ageing which is observed currently in most developed countries due to the increasing life expectancy. Given the variation that is observed in age related cognitive decline, a better understanding of the predictors and the determinants of cognitive performance and cognitive deterioration that is associated with ageing holds great importance, as it could set the basis for targeted interventions that could reduce the burden of dependence among older adults.

Cognitive performance is conceptualized as a set of different domains organized in a hierarchical order, with the bottom referring to basic sensory and perceptual abilities and the top referring to more complex functional abilities [1]. These different domains are not independent from one another as lower order functions enable the individual to receive, understand and remember information which will in turn be utilized through higher order functions of analysis and eventually synthesis. However, when examining different domains of cognition separately, there is variation in how these domains change with advancing age [2]. Generally, abilities such as executive function, memory and processing speed are reported to decline from midlife, while general knowledge remains more stable through ageing [3,4].

Processing speed has been described as an inherent ability similar to the clock speed of a computer in the sense that it dictates how long it will take for any cognitive task to be completed [5]. Consequently, processing speed is regarded a fundamental part of the cognitive system and it has been suggested that it is the reduction of this speed that mainly contributes to the impairment of cognitive functioning that is associated with age [6]. In fact, processing speed tends to be the strongest predictor of overall cognitive performance, loading highest in single factor solutions of cognitive ability [1]. However, little is known regarding the trajectories of processing speed performance through aging and their determinants, with a recent study by Bott et al. [7] associating a more stable course with genetic factors, lower inflammation and lifestyle characteristics such as physical activity.

The aim of this study is to identify latent groups of individuals with similar trajectories in processing speed as measured in the English Longitudinal Study of Aging (ELSA) dataset as well as to evaluate potential time-stable and time-varying determinants of each trajectory. Given the large sample and high quality of ELSA along with the significance of processing speed as a potential predictor of cognitive function in general, this analysis might provide valuable insight in the course of this specific cognitive domain through aging as well as age-related cognitive impairment in general.

## 2. Materials and Methods

### 2.1. Design and Setting

Data from a national and representative study of the English population, i.e., the English Longitudinal Study of Aging (ELSA), were used in this work [8]. ELSA is large-scale, panel study of 12,099 participants, aged ≥50 years, living in England, and is one of the longitudinal studies included in the ATHLOS project (an EU/HORIZON2020 funded project that aims to identify health trajectories and determinants of aging) [9]. Participants were recruited from households using a multistage stratified random probability design. Study’s participants were re-examined during the study’s course (2002–2012) in six-periodic examinations (waves), every 2 years, i.e., in 2004 (wave 2), 2006 (wave 3), 2008 (wave 4), 2010 (wave 5), and 2012 (wave 6).

All participants have given informed consent. Ethical approval for all the ELSA waves was granted from the National Research Ethics Service (MREC/01/2/91). Details of the ELSA study design, sample and data collection are available at the ELSA’s project website (https://www.elsa-project.ac.uk/, 18 April 2021).

### 2.2. Measurements 

#### 2.2.1. Processing Speed Assessment

Processing speed was assessed with a letter cancellation test. Participants had to identify and cross out as many of two target letters as possible from a page that included random letters in rows and columns in one minute [10]. The total number of correctly crossed out letters provided the measure of processing speed. The same test was performed in every wave that was included in the analysis.

#### 2.2.2. Baseline and Follow-Up Assessments

Participants’ baseline characteristics that were extracted from the ELSA dataset included sociodemographic and lifestyle factors. In particular, sex, age (in years), level of education (A-level or above rated as “high”, secondary education rated as medium and no qualifications rated as low), smoking habits (ever smoked, yes/no), level of physical activity on a weekly basis (rated as low, mild, moderate, and vigorous), marital status (never married, married, divorced or separated and widowed), history of smoking and level of wealth. Moreover, time-varying covariates measured in the 4 following waves included clinical characteristics, and particularly incidence of type 2 diabetes mellitus, any form of cardiovascular disease, as well as self-reported depression symptoms and sleep disturbances. These factors were studied here as they have been associated, directly or indirectly, with the main outcome of interest, cognitive function assessed in this study with processing speed performance. 

### 2.3. Statistical Analysis

Trajectories were estimated using group-based trajectory modeling (GBTM) [11]. This method fits a semi-parametric mixture model to longitudinal data using a maximum-likelihood method. The outcome variable was processing speed scores measured in waves 1 (baseline) to 5. Censored normal distribution model was selected as the outcome variable was handled as a continuous variable. The time metric of the model was the years an individual participated in the study (i.e., 1–8 years) as resulted by the number of waves (i.e., waves 2, 3, 4, and 5 correspond to 2, 4, 6, and 8 years in study, respectively). The number and polynomial shape of trajectories were selected based on the minimum value of the Bayesian Information Criterion (BIC) [12], after testing all potential models for two to four distinct groups for each age group before introducing covariates. The validity of the model was confirmed by calculating average posterior probability for each group as posterior probability higher than 70% indicates optimal fit. The selection of the model was made before introducing covariates. After the optimal model was chosen, time-stable and time-varying covariates were introduced simultaneously. Time stable covariates are baseline characteristics as described above that act as predictors of membership probability for each trajectory. Time-varying covariates are variables that may be positively or negatively correlated with the outcome variable within each trajectory. Categorical variables with more than two categories (education, level of physical activity and marital status) were introduced as dummy variables with one category set as reference. Level of wealth was available as the quintile of total household of each participant. The existing ordinal five-class variable describing participants’ wealth status was reclassified into a binary variable by merging classes 3, 4, and 5 (corresponding to the respective quintiles). Five time points were included in GBTM which were the 5 consecutive waves. The number of subjects varied in each wave. GBTM handles missing data by fitting the model using maximum likelihood estimation, based on the assumption that data are missing at random (MAR). Results are presented as odds ratio (OR) and 95% confidence interval (CI) for ORs for the time-stable covariates are estimated with a logistic function that is incorporated in GBTM package, accounting for for multiple correction and are interpreted as predictors of probability of belonging to each trajectory versus on that was set as baseline (Low). Statistical significance was considered at the level of a p-value < 0.05. The analyses were made using STATA Traj plug-in (Stata Corp., College Station, TX) [13]. 

## 3. Results

### 3.1. Baseline Characteristics of the Study Participants 

The sample at baseline consisted of 12.099 individuals. The majority of them were female (55.9%). Their mean age was 64.11 years at baseline (Range 50–94, SD = 10.26). 12.6% had received high level of education, 42.10% were of medium educational, while the remaining 45.3% had received low level education. The majority were married (70.58%), had a history of smoking (63%), and reported a mild level of physical activity (45.2%). Regarding the variables that were measured as time varying covariates, at baseline the majority did not report any history of cardiovascular incidents (85%), no depressive symptoms (80%), no history of diabetes mellitus (92.9%), and reported sleeping disturbances (57.38%). The mean processing speed score was 17.66 letters per minute at baseline. Table 1 summarizes the baseline characteristics of the study participants.

### 3.2. Trajectories of Processing Speed

**Figure 1:**
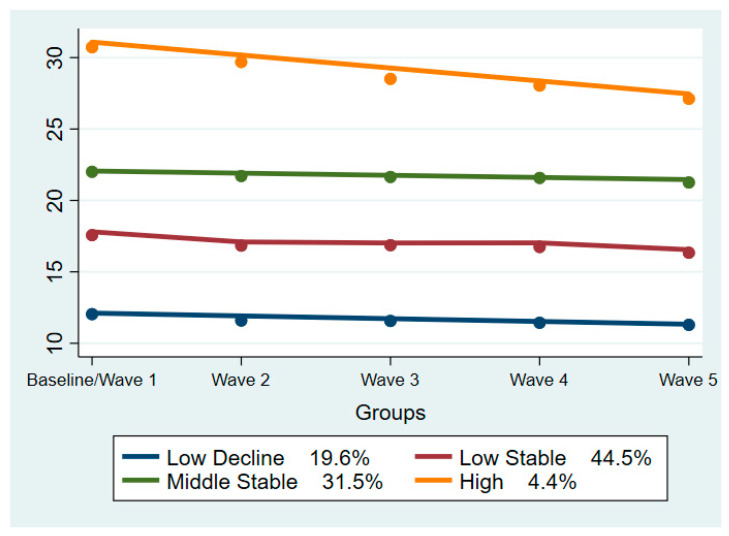
Trajectories of Processing Speed among the ELSA study participants. **Model BIC value:** −117,683.39.

A four-group model of first polynomial order for groups one, three and four and cubic for group two presented the best BIC values. More information about the selection of the model is provided in the Appendix A. Trajectory lines were named to describe their baseline scores and shapes as High, Middle/Stable, Low/Stable, and Low/Decline. Overall, 19.6% of the participants were classified the Low/Decline trajectory, 44.5% to the Low/Stable group, 31.5% to the Middle/Stable group, and 4.4% were classified to the High group. Figure 1 depicts trajectory lines. 

Table 2 summarizes the characteristics of time-stable covariates in our model. These parameters were used as predictors of the probability of belonging to each favorable trajectory, expressed as and odds ratio, compared to the probability of belonging to the “Low/Decline” group which was set as reference. Male sex was associated with a significantly lower probability of belonging to any favorable trajectory. The odds ratio was 0.42, 0.17, and 0.10 in the “Low/Stable”, “Middle/Stable”, and “High” groups, respectively. 

On the contrary, higher level of education was a strong a predictor of higher probability of belonging to all three favorable trajectories compared to the “Low/Decline” group. Both “Medium” and “High” level of education presented a statistically significant odds ratio favoring the probability of belonging to the favorable trajectories compared to the “Low” level of education that was set as reference.

Regarding marital status, the category “widowed” was the one that was associated with lower probability of belonging to any of the favorable groups. However, the association was only significant in the “High” vs “Low/Decline” group comparison (*p*-value = 0.003, OR = 0.43). On the contrary, the categories “Separated or Divorced” and “Married” both presented an increased likelihood of belonging to the favorable groups compared to the category “not married” that was set as reference.

As for level of self-reported physical activity, the category that was associated with most beneficial results was the “Mild”, which was associated with significantly increased probability of belonging to all the favorable groups. On the other hand, both “Moderate” and “Vigorous” levels of physical activity were consistently associated with less likelihood of belonging to any of the favorable trajectories compared to the “No Physical Activity” category that was used as reference.

### 3.3. Processing Speed Trajectories in Relation to Chronic Illnesses 

Incidence of cardiovascular events was inversely associated with the scores of processing speed in all trajectories. This association was statistically significant in all groups except for the “Low/Decline”. The same relationship was also observed between processing speed and presence of diabetes mellitus and was significant in all the groups except for the “High”. The presence of depressive symptoms was also associated with lower scores of processing speed, but this association was only significant in the “Low/Decline” and “Low/Stable” trajectories. Finally, sleeping disturbances were negatively correlated with processing speed scores in the “Low/Decline” trajectory, but positively in the “High” group with a p-value that indicates statistical significance (<0.001 and 0.034 respectively). Table 3 summarizes the time-varying covariates across the four trajectories of processing speed scores, in relation to cardiovascular disease, depressive symptomatology, sleeping disturbances, and diabetes mellitus.

## 4. Discussion

The present study identified four latent groups of processing speed scores trajectories in our sample. All latent groups presented a slight declining trend over the years. The trajectory with the steepest decreasing trend was the “High” one. This might be mainly attributed to the fact that it is the trajectory that consists of fewer individuals (4.3% of the total sample), so a more rapid decline in some participants will have a greater impact in the overall performance of the group. Previous studies investigating latent classes of cognitive performance assessed with various neuropsychological tests have also identified four or three trajectories [14,15,16] with similar shapes. The slightly less decreasing and more stable trend observed in the trajectories in the present study might be explained by the shorter duration of observation of our study, as cognitive decline is a slow process that might require a longer period to be reflected or the different course of processing speed compared to other cognitive domains. However, what is common in all these studies, including ours, is that the trajectory lines do not mix and higher performance at baseline predicts higher performance also at the end of study, depicting that cognitive performance with advanced age is at some degree dependent on the mental capacity that an individual has at their prime.

Level of education was found to play an important role in the probability of belonging to a favorable trajectory. In particular, medium level of education was a predictor of greater likelihood to belong to all three favorable trajectories, compared to the low level that was set as reference, with a p-value that indicates statistical significance (<0.001 in all three groups). What is interesting is that the OR, that reflects the magnitude of this association, presented an increasing trend from the lowest to the highest trajectory (1.88 in the Low/Stable, 3.48 Middle/Stable, 4.04 in the High). The same trend was observed in the effect of the High level of education, differing but at an even greater scale. The OR of the High level of education versus the low that was set as reference was 2.267, 8.87, 12.39 in the Low/Stable, Middle/Stable and High group respectively. Conclusively, based on these findings, it can be hypothesized that education is a strong predictor of predictor of better cognitive performance and the magnitude of this association increases not only with the increasing level of education but also with the higher cognitive performance. These findings are in line with a recent study be Ferraro et al. [17] where higher number of years of education was associated with better physical, functional and cognitive performance. Education is consistently reported a protective factor for cognitive impairment and dementia [18,19]. The findings of the current study confirm this beneficial effect and highlight the importance of education as a potential modifiable factor that can reduce the burden of cognitive impairment across the ageing population.

Marital status also presented a consistently significant association with our outcome of interest. The categories “Married” and “Divorced or Separated” presented a significantly higher probability of belonging to all three favorable trajectories (except for “High” group with a p value of 0.075 and 0.055 respectively) compared to the “Never Married” category that was set as reference. On the contrary, the category “Widowed” was associated with lower probability of belonging to any favorable group compared to the reference category, which was only significant in the “High” group. Previous studies have also reported the association between widowhood and greater risk for cognitive decline [20] as well as a greater risk for cognitive impairment in single adults compared to those cohabiting with a partner [21]. The findings can be explained by viewing marital status’s effect as part of the overall effect that social life and social isolation have on the cognitive function of older adults [22]. The exact mechanisms behind these effects are not clear, but since this a real-world study any consistent association is worth highlighting as it might set the basis for future research.

The level of self-reported physical activity also presented an interesting association with the trajectories of processing speed in our cohort. “Mild” level of physical activity presented a consistently significant association with greater probability of belonging to any of the favorable trajectories compared to the “no physical activity” that was set as reference. On the contrary, the association between the categories of “Moderate” and “Vigorous” physical activity was just as consistent but inverse, interpreted as less probability of belonging to any of the three favorable groups for both these categories. Physical activity is mainly regarded as a protective factor for the cognitive function of older adults [23]. However, there is no clear consensus regarding its characteristics (type, frequency, duration, intensity) that seem to provide the most beneficial results. In fact, it has been suggested that high intensity exercise might even worsen cognitive performance [24]. From that view, the findings of our study are partly in line with published literature, however based on these alone no further implications can be made.

Finally, level of household wealth also presented a consistent association, as higher level of wealth was a significant predictor of belonging to all three favorable trajectories. This does not come as a surprise as higher socioeconomic status has been associated with better cognitive function in older adults [25] and this is confirmed in the present study.

Regarding time-varying covariates, incidence of cardiovascular events presented a statistically significant negative correlation with processing speed scores across all groups except for the “Low/Decline” where it presented a marginal p-value of 0.094. There is significant evidence in published literature linking cardiovascular fitness and cognitive performance [26]. Aside from vascular dementia where there is the obvious association of cardiovascular health with brain perfusion and cognitive performance, it has been shown that managing cardiovascular risk factors might decrease the risk of other forms of dementia such as Alzheimer’s disease [27]. Although the incidence of diabetes mellitus follows the same principles with the rest of cardiovascular risk factors regarding its association with cognitive function, it seems to hold an exquisite importance [28]. This was reflected in the current study too, where incidence of diabetes mellitus was significantly associated with lower processing speed scores across all trajectories, except for the “High”, where the same association was close to statistical significance with a p-value of 0.062. These findings are in line with those of the study of Marseglia et al. [29] where in a sample of 793 adults over 50 years of age, those with diabetes presented a steeper decline in perceptual speed. Conclusively, based on our findings in addition to what is already known, management of cardiovascular risk factors and especially of diabetes mellitus is an essential target for interventions in order to decrease the burden of cognitive decline in older adults.

Presence of depressive symptoms showed a negative correlation with processing speed scores across all trajectories, which was statistically significant in the two lower groups. Cognitive impairment is mentioned as core feature of depression and not an epiphenomenon [30]. Moreover, it is quite common depressive symptoms (such as memory problems) to be mistakenly attributed to cognitive impairment. Although one cannot establish a clear relationship of cause and effect between cognitive decline and depression, our study confirms the association between these two conditions. The lack of a consistently statistically significant relationship in more trajectories may be partly due to the fact that presence of depressive symptoms was self-reported and not accurately measured with specific neuropsychological scales. Nevertheless, when thinking of targeted interventions, depression should be considered as a potential modifiable factor. Finally, self-reported sleep disturbances did not present a consistent relationship with processing speed scores.

The results of this study should be viewed in the light of its limitations. Firstly, the participants were all living in England, weakening the generalizability of the findings in populations from other countries or continents. Moreover, information on certain variables, such as depressive symptoms, was self-reported and greater detail regarding the duration of these symptoms was not available. Additionally, all participants were included in the analysis. This might result in a degree of heterogeneity, as some of the participants might have already presented mild cognitive impairment or dementia at the beginning of the study, differing significantly in terms of cognitive decline over the years from the rest of the participants. However, despite its limitations this study was based on real world data from a big sample of high-quality longitudinal study and the analyses were made utilizing unbiased techniques. Moreover, this is the first analysis to our knowledge investigating the trajectories of processing speed using the GBTM. Taking these into account, our findings provide useful insight in the course of the processing speed through aging, which in addition to similar analyses of different cognitive domains [9], strengthen our knowledge about the epidemiology of age-related cognitive decline in general.

## 5. Conclusions

Four latent groups of processing speed were identified in our study. Level of education was a strong predictor of a more favorable trajectory, whereas the presence of cardiovascular diseases and diabetes mellitus were associated with lower processing scores within groups. Depressive symptoms also presented a significant association with lower scores in some groups. The aforementioned factors might represent valid targets for interventions, while future studies might elucidate the significance of the impact of physical activity and social factors, such as marital status, on the cognitive function of older adults. 

## Figures and Tables

**Table 1:** Baseline characteristics of the ELSA study participants that included in the present analyses.

Characteristic	Overall N = 12099
**Marital Status**	
Single	575 (4.75%)
Married	8539 (70.58%)
Separated, Divorced	1034 (8.55%)
Widowed	1951 (16.13%)
**Sex, males**	5335 (44.1%)
**Education**	
Low	5008 (41.39%)
Medium	4653 (38.46%)
High	1388 (11.47%)
NA values	1040 (8.5%)
**Physical activity level**	
No Physical Activity	1835 (16.9%)
Mild	4909 (45.22%)
Moderate	2483 (22.87%)
Vigorous	654 (6.02%)
NA values	975 (9%)
**Ever smoked, yes**	7623 (63%)
**History of cardiovascular disease, yes**	1804 (15.9%)
**History of Diabetes, yes**	866 (7.16%)
**History of Depressive Symptoms, yes**	1923 (15.9%)
**History of Sleeping Disturbances, yes**	6942 (57.38%)

**Table 2:** Results from logistic regression models that evaluated factors associated with processing speed in the ELSA study participants.

Group	Parameter	OR	Lower 95%CL	Upper 95%CL	P-value
**Low/Stable vs Low/Decline**	Male vs Female	0.425	0.357	0.505	<0.001
	Education Level				
	High vs Low	2.672	1.846	3.868	<0.001
	Medium vs Low	1.879	1.577	2.239	<0.001
	Marrital Status				
	Widowed vs Never Married	0.816	0.588	1.134	0.226
	Separated or Divorced vs Never Married	2.113	1.430	3.124	<0.001
	Married vs Never Married	1.848	1.364	2.504	<0.001
	Level of Physical Activity (PA)				
	Vigorous vs No PA	0.333	0.247	0.449	<0.001
	Moderate vs No PA	0.805	0.651	0.995	0.045
	Mild vs No PA	1.569	1.287	1.913	<0.001
	High Wealth	1.716	1.463	2.013	<0.001
	Ever Smoked	1.038	0.881	1.224	0.655
**Middle/Stable vs Low/Decline**	Male vs Female	0.171	0.143	0.204	<0.001
	Education Level				
	High vs Low	8.869	6.283	12.519	<0.001
	Medium vs Low	3.485	2.923	4.155	<0.001
	Marrital Status				
	Widowed vs Never Married	0.771	0.528	1.127	0.180
	Separated or Divorced vs Never Married	3.747	2.456	5.717	<0.001
	Married vs Never Married	3.097	2.189	4.382	<0.001
	Level of Physical Activity (PA)				
	Vigorous vs No PA	0.158	0.106	0.236	<0.001
	Moderate vs No PA	0.733	0.595	0.902	<0.003
	Mild vs No PA	1.527	1.260	1.850	<0.001
	High Wealth	2.381	2.018	2.808	<0.001
	Ever Smoked	0.938	0.798	1.104	0.442
**High vs Low/Decline**	Male vs Female	0.100	0.075	0.134	<0.001
	Education Level				
	High vs Low	12.389	7.923	19.373	<0.001
	Medium vs Low	4.049	3.064	5.351	<0.001
	Marrital Status				
	Widowed vs Never Married	0.430	0.248	0.744	0.003
	Separated or Divorced vs Never Married	1.802	0.988	3.286	0.055
	Married vs Never Married	1.540	0.958	2.477	0.075
	Level of Physical Activity (PA)				
	Vigorous vs No PA	0.119	0.046	0.308	<0.001
	Moderate vs No PA	0.783	0.554	1.107	0.167
	Mild vs No PA	1.710	1.277	2.290	<0.001
	High Wealth	1.963	1.496	2.577	<0.001
	Ever Smoked	1.062	0.826	1.366	0.639

**Table 3:** Time-varying covariates of processing speed categories, regarding various health outcomes.

Group	Outcome	Beta Estimate	Standard Error	p-value
**Low/Decline**	Cardiovascular disease	−0.354	0.211	0.094
	Depressive Symptoms	−0.967	0.161	<0.001
	Sleeping Disturbances	−0.461	0.132	<0.001
	Diabetes Mellitus	−0.433	0.205	0.035
**Low /Stable**	Cardiovascular disease	−0.871	0.154	<0.001
	Depressive Symptoms	−0.542	0.112	<0.001
	Sleeping Disturbances	−0.121	0.082	0.139
	Diabetes Mellitus	−0.636	0.142	<0.001
**Middle/Stable**	Cardiovascular disease	−0.819	0.175	<0.001
	Depressive Symptoms	−0.150	0.137	0.276
	Sleeping Disturbances	−0.070	0.090	0.435
	Diabetes Mellitus	−0.672	0.174	<0.001
**High**	Cardiovascular disease	−5.214	0.998	<0.001
	Depressive Symptoms	0.507	0.404	0.209
	Sleeping Disturbances	0.577	0.272	0.034
	Diabetes Mellitus	−0.939	0.504	0.062

## Appendix A

### Choice of Model

The choice of model was based on the best BIC value. The formula for estimating BIC values in GBTM is BIC = log(L)−0.5klog(N ), and the choice of the model with the highest BIC value is suggested [11]. Alternative models were also tested, but did not differ significantly in the shapes and percentages of the trajectories. After model selection, average posterior probability for each group is estimated to test the fitness of the model. Average posterior probability was calculated and was above 70% percent in all groups, indicating a good fit. Due to one group consisting of less than 5% of the sample, models with three trajectories were also explored. However, they kept producing a similar “high” trajectory consisting of a slightly higher percentage of the sample (7%). Based on these, we decided that the best fitting model was the one that was selected.

**Table A1:** **Models**.

Groups	Polynomial Orders	BIC Value
4	1311	−117683
4	1312	−117686
4	1111	−117686
4	1411	−117688
4	1112	−117688
4	2311	−117688
..	..	..
..	..	..
3	111	−118358

**Table A2:** Average Posterior Probability.

Group	APP
**1**	**0.82**
**2**	**0.77**
**3**	**0.82**
**4**	**0.89**

**Table A3:** Participants Per Wave.

Wave	Number of Participants
**1**	12,099
**2**	9432
**3**	9741
**4**	11,050
**5**	10,317

**Table A4:** Distribution of Determinants Across Trajectories.

Characteristic	Trajectory			
Marital Status	Low Decline	Middle Decline	Middle Stable	High
Single	219 (2%)	268 (2.43%)	51 (0.46%)	21 (0.19%)
Married	1762 (16%)	4508 (40.91%)	1100 (9.98%)	164 (1.49%)
Separated, Divorced	272 (2.47%)	561 (5.1%)	176 (1.6%)	19 (0.17%)
Widowed	516 (4.68%)	1030 (9.35%)	309 (2.8%)	44 (0.4%)
**Sex, males**	1749 (15.87%)	2791 (25.33%)	436 (3.96%)	54 (0.5%)
**Education**				
Low	1635 (16/27%)	2497 (24.85%)	486 (4.84%)	83 (0.83%)
Medium	800 (7.26%)	2541 (23.06%)	678 (6.15%)	107 (0.97%)
High	116 (1.05%)	762 (6.91%)	302 (2.74%)	41 (0.37%)
**Physical activity level**				
No Physical Activity				
Mild	903 (8.19%)	2676 (24.28%)	810 (7.35%)	119 (1.08%)
Moderate	671 (6.09%)	1210 (10.1%)	387 (3.51%)	54 (0.5%)
Vigorous	339 (3.08%)	222 (2.01%)	52 (0.47%)	3 (0.03%)
NA values				
**Ever smoked, yes**	1915 (17.38%)	4103 (37.23%)	946 (8.58%)	149 (1.35%)
**CVD, yes**	562 (5.1%)	926 (8.4%)	192 (1.72%)	25 (0.23%)
**Diabetes, yes**	265 (2.41%)	452 (4.1%)	88 (0.8%)	13 (0.12%)
**Depressive Symptoms, yes**	601 (5.54%)	949 (8.75%)	227 (2.09%)	22 (0.2%)
**Sleeping Disturbances, yes**	1554 (14.3%)	3728 (34.31%)	967 (8.9%)	161 (1.48%)

## References

[1] Harvey P.D. (2019). Domains of cognition and their assessment. Dialogues Clin. Neurosci..

[2] Gale C.R., Allerhand M., Sayer A.A., Cooper C., Deary I.J. (2014). The dynamic relationship between cognitive function and walking speed: The English Longitudinal Study of Ageing. Age.

[3] Park D.C., Reuter-Lorenz P. (2009). The adaptive brain: Aging and neurocognitive scaffolding. Annu. Rev. Psychol..

[4] Singh-Manoux A., Kivimaki M., Glymour M.M., Elbaz A., Berr C., Ebmeier K.P., Ferrie J.E., Dugravot A. (2012). Timing of onset of cognitive decline: Results from Whitehall II prospective cohort study. BMJ.

[5] Ridderinkhof K.R., van der Molen M.W. (1997). Mental resources, processing speed, and inhibitory control: A developmental perspective. Biol. Psychol..

[6] Salthouse T.A. (1996). The processing-speed theory of adult age differences in cognition. Psychol. Rev..

[7] Bott N.T., Bettcher B.M., Yokoyama J.S., Frazier D.T., Wynn M., Karydas A., Yaffe K., Kramer J.H. (2017). Youthful Processing Speed in Older Adults: Genetic, Biological, and Behavioral Predictors of Cognitive Processing Speed Trajectories in Aging. Front. Aging Neurosci..

[8] Steptoe A., Breeze E., Banks J., Nazroo J. (2013). Cohort Profile: The English Longitudinal Study of Ageing. Int. J. Epidemiol..

[9] Sanchez-Niubo A., Egea-Cortés L., Olaya B., Caballero F.F., Ayuso-Mateos J.L., Prina M., Bobak M., Arndt H., Tobiasz-Adamczyk B., Pająk A. (2019). The Ageing Trajectories of Health-Longitudinal Opportunities and Synergies (ATHLOS) project. Int. J. Epidemiol..

[10] de la Fuente J., Hjelmborg J., Wod M., de la Torre-Luque A., Caballero F.F., Christensen K., Ayuso-Mateos J.L. (2019). Longitudinal Associations of Sensory and Cognitive Functioning: A Structural Equation Modeling Approach. J. Gerontol. B Psychol. Sci. Soc. Sci..

[11] Nagin D. (2005). Group-Based Modeling of Development.

[12] Nagin D.S., Odgers C.L. (2010). Group-Based Trajectory Modeling in Clinical Research. Annu. Rev. Clin. Psychol..

[13] Jones B.L., Nagin D.S. (2013). A Note on a Stata plugin for estimating group-based trajectory models. Sociol. Methods Res..

[14] Olaya B., Bobak M., Haro J.M., Demakakos P. (2017). Trajectories of Verbal Episodic Memory in Middle-Aged and Older Adults: Evidence from the English Longitudinal Study of Ageing. J. Am. Geriatr. Soc..

[15] Zahodne L.B., Wall M.M., Schupf N., Mayeux R., Manly J.J., Stern Y., Brickman A.M. (2015). Late-life memory trajectories in relation to incident dementia and regional brain atrophy. J. Neurol..

[16] Terrera G.M., Brayne C., Matthews F., the CC75C Study Collaboration Group (2010). One size fits all? Why we need more sophisticated analytical methods in the explanation of trajectories of cognition in older age and their potential risk factors. Int. Psychogeriatr..

[17] Ferraro O.E., Guaita A., Villani S. (2021). Cognitive, physical and disability trajectories in community-dwelling elderly people [published online ahead of print, 2021 Feb 16]. Aging Clin. Exp. Res..

[18] McDowell I., Xi G., Lindsay J., Tierney M. (2007). Mapping the connections between education and dementia. J. Clin. Exp. Neuropsychol..

[19] Schmand B., Smit J., Lindeboom J., Smits C., Hooijer C., Jonker C., Deelman B. (1997). Low education is a genuine risk factor for accelerated memory decline and dementia. J. Clin. Epidemiol..

[20] Biddle K.D., Jacobs H.I.L., d’Oleire Uquillas F., Zide B.S., Kirn D.R., Properzi M.R., Rentz D.M., Johnson K.A., Sperling R.A., Donovan N.J. (2020). Associations of Widowhood and β-Amyloid With Cognitive Decline in Cognitively Unimpaired Older Adults. JAMA Netw. Open.

[21] Håkansson K., Rovio S., Helkala E.-L., Vilska A.-R., Winblad B., Soininen H., Nissinen A., Mohammed A.H., Kivipelto M. (2009). Association between mid-life marital status and cognitive function in later life: Population based cohort study. BMJ.

[22] Evans I.E., Martyr A., Collins R., Brayne C., Clare L. (2019). Social Isolation and Cognitive Function in Later Life: A Systematic Review and Meta-Analysis. J. Alzheimer’s Dis..

[23] Northey J.M., Cherbuin N., Pumpa K.L., Smee D.J., Rattray B. (2018). Exercise interventions for cognitive function in adults older than 50: A systematic review with meta-analysis. Br. J. Sports Med..

[24] Chang H., Kim K., Jung Y.-J., Kato M. (2017). Effects of acute high-intensity resistance exercise on cognitive function and oxygenation in prefrontal cortex. J. Exerc. Nutr. Biochem..

[25] Zhang M., Gale S.D., Erickson L.D., Brown B.L., Woody P., Hedges D.W. (2015). Cognitive function in older adults according to current socioeconomic status. Neuropsychol. Dev. Cogn. B Aging Neuropsychol. Cogn..

[26] Stuckenschneider T., Askew C.D., Rüdiger S., Polidori M.C., Abeln V., Vogt T., Krome A., Rikkert M.O., Lawlor B., Schneider S. (2018). Cardiorespiratory Fitness and Cognitive Function are Positively Related Among Participants with Mild and Subjective Cognitive Impairment. J. Alzheimer’s Dis..

[27] Fillit H., Nash D.T., Rundek T., Zuckerman A. (2008). Cardiovascular risk factors and dementia. Am. J. Geriatr. Pharmacother..

[28] Moheet A., Mangia S., Seaquist E.R. (2015). Impact of diabetes on cognitive function and brain structure. Ann. N. Y. Acad. Sci..

[29] Marseglia A., Aslan A.K.D., Fratiglioni L., Santoni G., Pedersen N.L., Xu W. (2018). Cognitive Trajectories of Older Adults With Prediabetes and Diabetes: A Population-Based Cohort Study. J. Gerontol. Ser. A Biol. Sci. Med. Sci..

[30] Rock P.L., Roiser J.P., Riedel W.J., Blackwell A.D. (2014). Cognitive impairment in depression: A systematic review and meta-analysis. Psychol. Med..

